# The Emerging Interplay Between Recirculating and Tissue-Resident Memory T Cells in Cancer Immunity: Lessons Learned From PD-1/PD-L1 Blockade Therapy and Remaining Gaps

**DOI:** 10.3389/fimmu.2021.755304

**Published:** 2021-11-16

**Authors:** Silvia Gitto, Ambra Natalini, Fabrizio Antonangeli, Francesca Di Rosa

**Affiliations:** ^1^ Institute of Molecular Biology and Pathology, National Research Council of Italy (CNR), Rome, Italy; ^2^ Department of Molecular Medicine, University of Rome “Sapienza”, Rome, Italy

**Keywords:** immune checkpoint blockade, memory T cells, TRM, bone marrow, anti-tumor immunity

## Abstract

Remarkable progress has been made in the field of anti-tumor immunity, nevertheless many questions are still open. Thus, even though memory T cells have been implicated in long-term anti-tumor protection, particularly in prevention of cancer recurrence, the bases of their variable effectiveness in tumor patients are poorly understood. Two types of memory T cells have been described according to their traffic pathways: recirculating and tissue-resident memory T cells. Recirculating tumor-specific memory T cells are found in the cell infiltrate of solid tumors, in the lymph and in the peripheral blood, and they constantly migrate in and out of lymph nodes, spleen, and bone marrow. Tissue-resident tumor-specific memory T cells (TRM) permanently reside in the tumor, providing local protection.

Anti-PD-1/PD-L1, a type of immune checkpoint blockade (ICB) therapy, can considerably re-invigorate T cell response and lead to successful tumor control, even in patients at advanced stages. Indeed, ICB has led to unprecedented successes against many types of cancers, starting a ground-breaking revolution in tumor therapy. Unfortunately, not all patients are responsive to such treatment, thus further improvements are urgently needed. The mechanisms underlying resistance to ICB are still largely unknown. A better knowledge of the dynamics of the immune response driven by the two types of memory T cells before and after anti-PD-1/PD-L1 would provide important insights on the variability of the outcomes. This would be instrumental to design new treatments to overcome resistance.

Here we provide an overview of T cell contribution to immunity against solid tumors, focusing on memory T cells. We summarize recent evidence on the involvement of recirculating memory T cells and TRM in anti-PD-1/PD-L1-elicited antitumor immunity, outline the open questions in the field, and propose that a synergic action of the two types of memory T cells is required to achieve a full response. We argue that a T-centric vision focused on the specific roles and the possible interplay between TRM and recirculating memory T cells will lead to a better understanding of anti-PD-1/PD-L1 mechanism of action, and provide new tools for improving ICB therapeutic strategy.

## Introduction

**Figure 1:**
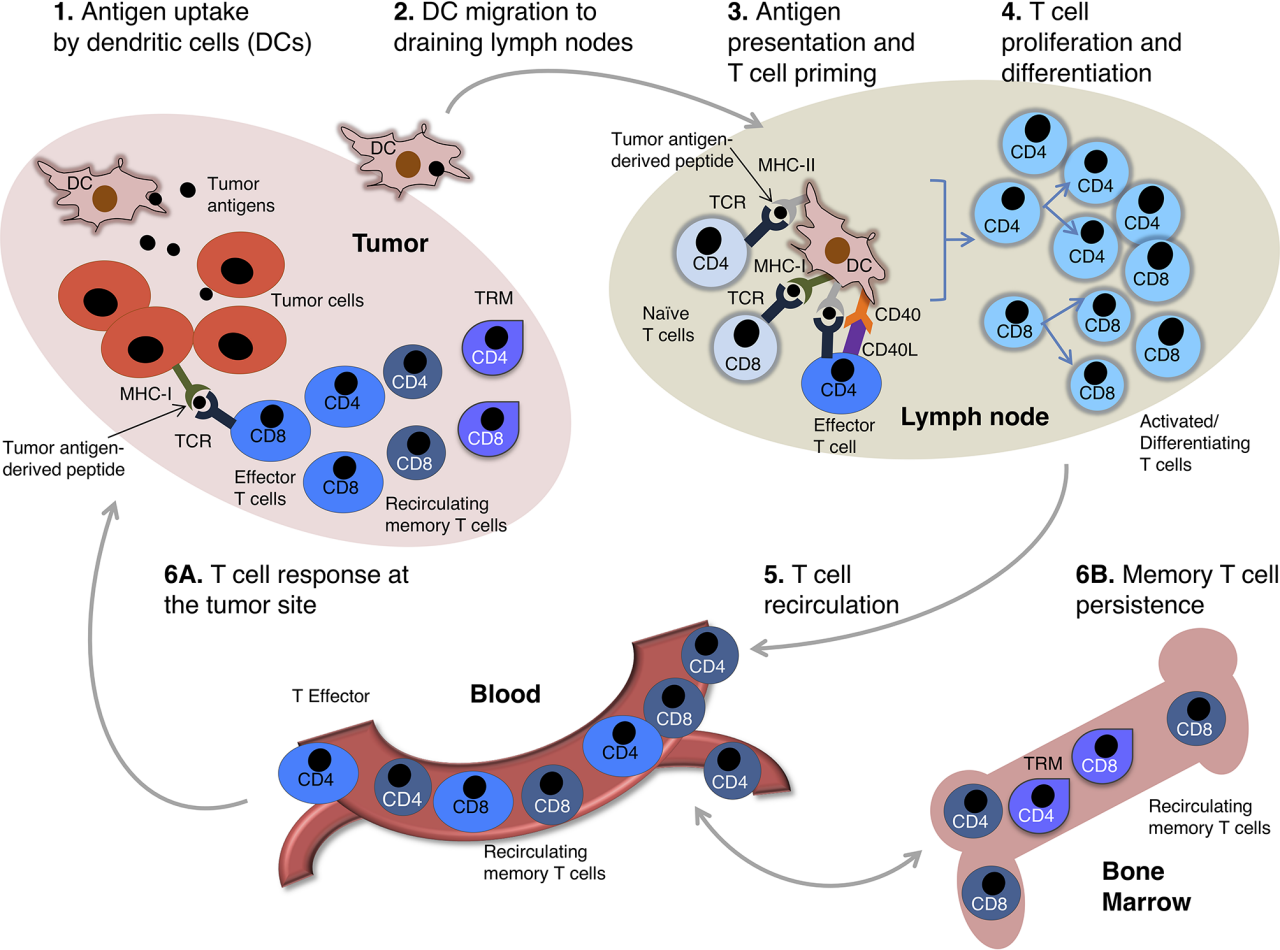
The circuit of anti-tumor T cell immunity. A scheme of anti-tumor T cell response is depicted. Intratumoral DCs uptake antigens released by damaged/dying tumor cells (**1**). DCs get activated and migrate to tumor draining lymph nodes (LNs) (**2**) wherein they present tumor antigen-derived peptides in the context of MHC molecules, plus costimulatory signals, to naïve tumor-specific T cells. Tumor antigen-derived peptides presented in MHC-II and MHC-I molecules are recognized by CD4 and CD8 T cells, respectively. Activated CD4 T cells differentiating into effector cells up-regulate CD40L and provide help for naïve CD8 T cells by modulating DC costimulatory capability (so-called DC “licensing”, mediated by CD40L-CD40 interaction) (1–4). Licensed DCs are enabled to provide full costimulation for naïve CD8 T cell priming (**3**) (note that DC-provided costimulatory signals for T cell priming are not depicted for simplicity). Activated tumor-specific CD4 and CD8 T cells proliferate and differentiate (**4**), and migrate out of LNs into the blood stream (**5**). The differentiated progeny includes effector and memory T cells, which extravasate from the blood and enter the tumor sites (**6A**). Tissue-resident memory T cells (TRM) are non-migratory cells that persist in the tumor (5), whereas recirculating memory T cells go back to circulation and migrate all over the body (6). They preferentially accumulate in the bone marrow (BM), a key organ for long-term memory T cell maintenance (7, 8) (**6B**).

**Figure 2:**
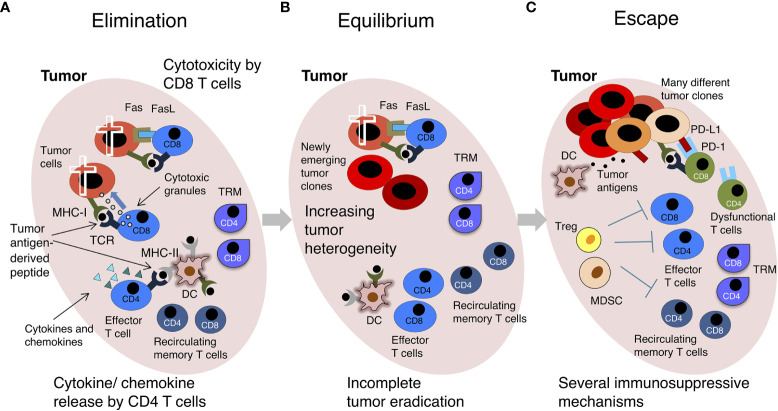
The “Cancer Immunoediting” concept: effector versus exhausted T cells. A scheme of the 3 phases of “Cancer Immunoediting” is represented, with emphasis on T cells. **(A)** Elimination. The tumor has low heterogeneity. Effector CD8 T triggered by recognition of tumor-derived peptides in the context of MHC-I molecules on the surface of tumor cells kill these cells by release of secretory granules (containing perforin, granzymes, etc.) and FasL/Fas interaction. Effector CD4 T cells triggered by recognition of tumor-derived peptides in the context of MHC-II molecules on the surface of DCs and other tumor-infiltrating immune cells release cytokines and chemokines. **(B)** Equilibrium. There is moderate intratumoral heterogeneity, and tumor mass contains some genetically different tumor clones. TILs control tumor growth without inducing tumor regression. **(C)** Escape. Tumor is genetically unstable and highly heterogeneous. The immune cell infiltrate contains a few effector T cells and high levels of regulatory T cells (Tregs) and Myeloid Derived Suppressor Cells (MDSCs), which contribute to create an immunosuppressive environment. DCs do not effectively present tumor-derived antigens and T cell-inhibitory circuits are dominant, for example that induced by the interaction between PD-L1^+^ tumor cells and PD-1^+^ T cells. Most intratumoral T cells are dysfunctional. The tumor grows. For simplicity, only some cells are depicted. See text and original reference (9) for more details.

T cells are major players of anti-tumoral immunity. During the induction phase of an adaptive immune response against cancer cells, dendritic cells (DCs) uptake tumor antigens released by damaged/dying tumor cells and migrate to secondary lymphoid organs, such as tumor-draining lymph nodes (LNs). In these organs DCs present tumor antigen-derived peptides in the context of Major Histocompatibility Complex (MHC) molecules of class I (MHC-I) and class II (MHC-II) to naïve CD8 and CD4 T cells, respectively. MHC-peptide complexes and costimulatory molecules expressed by DCs jointly lead to T cell priming (**Figure 1**). In most cases, effective naïve CD8 T cell priming requires CD4 T cell help. This is mediated *via* CD40L^+^ CD4 T cell interaction with CD40^+^ DCs. The DCs are thus “licensed” and can provide all the costimulatory signals needed for naïve CD8 T cell priming (1–4) (**Figure 1**). Primed CD4 and CD8 T cells proliferate and generate a progeny of short-lived effector and long-lived memory cells that migrate out of LNs. Effector T cells enter the tumor bed, recognize tumor-antigens in this site and display their protective function. Specifically, cytotoxic CD8 T cells kill tumor cells *via* degranulation of secretory granules or activation of the Fas/FasL molecular pathway, whereas CD4 T cells provide help for CD8 T cell stimulation, and produce pro-inflammatory cytokines (e.g. TNF-α, IFN-γ, etc.) and chemokines that attract further effector T cells into the tumor (**Figures 1** and **2A**). After the acute phase of an immune response, antigen is cleared, effector T cells die, and a few memory T cells remain over long time in the blood and in tissue reservoirs, including the bone marrow (BM) (7, 8) (**Figure 1**). Memory T cells are more abundant than their naïve precursors, and are poised to proliferate, differentiate and display prompt effector function upon secondary stimulation, resulting in a more rapid and efficient antigen elimination than in the primary response. Unfortunately, T cell response against cancer cells does not always lead to antigen clearance. Tumor-bearing patients typically present with a chronic immune response, characterized by persisting antigen, and deficient and/or dysfunctional anti-tumor T cells (**Figures 1** and **2**).

The mechanisms underlying qualitatively and quantitatively effective T cell memory are largely unclear. The most accepted view is that antigen-specific memory T cells are maintained for years by a fine equilibrium between quiescence and self-renewal. It has been proposed that a duality of BM niches supports memory T cells persistence over time, without consuming their proliferative potential (8). Two major types of memory T cells have been recently distinguished according to their migratory behavior, i.e. recirculating memory T cells and tissue-resident memory T cells (TRM) (**Figure 1**). Recirculating memory T cells include central memory T cells (TCM) and effector memory T cells (TEM), that are discriminated based on the expression of CCR7, a lymph node (LN) homing receptor (10). Recirculating memory T cells migrate through the blood and the lymph, and patrol the whole body. In contrast, TRM are found in peripheral sites, in disconnection from circulation, thus these cells provide local and/or tissue-wide protection (5, 6). Despite increasing knowledge on T cell memory, many gaps still remain to be filled, especially in respect to TRM, given the relatively recent discovery of these cells. For example, while recirculating memory T cells with phenotype and transcriptional signature similar to hematopoietic stem cells (T stem cell memory cells, TSCM) have been implicated in long-term memory (11–13), a similar TRM subset has been only partially characterized (14, 15). It is also unknown whether TRM residing in different tissues have a diverse longevity (6). Furthermore, many questions about the role of TRM in anti-tumoral immunity, and their interplay with recirculating memory T cells, remain open.

Anti-tumor T cell response takes place in the context of a complex relationship between a growing tumor and the immune system. According to the concept of “Cancer Immunoediting”, this relationship is a multi-step process including three phases, i.e. elimination, equilibrium and escape (the so-called three “Es” of cancer immunoediting) (9) (**Figure 2**). The elimination phase is characterized by the physical deletion of MHC-I^+^ tumor cells by infiltrating effector CD8 T cells, which are triggered by recognition of tumor antigen-derived peptides in the context of MHC-I. Similarly, tumor-specific effector CD4 T cells can be triggered to release pro-inflammatory cytokines and chemokines by antigen-MHC-II complexes presented by DCs in the tumor microenvironment (TME). Intratumoral DCs further emphasize anti-tumor immunity by restimulating effector T cells locally (**Figure 2A**). In the equilibrium phase there is a dynamic interplay between genetically heterogeneous tumor cells, and the immune cell infiltrate. Tumor-infiltrating lymphocytes (TILs) exert a containing function, without eradicating the tumor. In fact, some tumor cell variants acquire mutations that give them a survival advantage and/or enable them to resist to the antitumoral immune response. Selective killing of tumor cells by functional effector T cells contributes to tumor editing, a sort of “Darwinian selection” that gives advantages to tumor cells able to avoid immune cell recognition or killing (16) (**Figure 2B**). The escape phase is characterized by the expansion of tumor cell variants, which have often lost their sensitivity to the immune attack through several genetic and epigenetic alterations. At this stage, the TME is typically immunosuppressive, e.g. enriched with regulatory T cells (Tregs), myeloid-derived suppressor cells (MDSCs), etc., and the infiltrating tumor-specific CD4 and CD8 T cells are often dysfunctional (**Figure 2C**). In addition to Tregs and MDSCs, several players of cancer-initiated negative circuits have been identified in advanced cancer-bearing individuals, for example γδ T cells, macrophages and neutrophils can cooperate in suppressing CD8 T cells (17). Dysfunctional anti-tumor T cells in tumor-bearing patients are often considered the equivalent of “exhausted” anti-viral T cells in mice chronically infected with lymphocytic choriomeningitis virus (LCMV) (18–20). Typically, exhausted T cells are exposed to high dose/persisting antigen and have impaired effector function, nevertheless the concept of T cell-exhaustion has been differently defined in diverse contexts, generating potential misunderstanding (21). We will use it here to indicate the complex phenotype of dysfunctional tumor-specific T cells in patients with clinically evident tumors (**Figure 2C**).

When a patient presents with a clinically evident tumor, the above-described tumor-host interaction is mostly in the escape phase (9). Anti-cancer T cells are inhibited by so-called immune checkpoints, i.e. negative feedback pathways that normally prevent excessive activation in chronic immune responses. A series of inhibitory receptor/ligand pairs controlling T cell response have been described, e.g., PD-1/PD-L1, CTLA4/B7, LAG-3/MHCII, TIM-3/Galectin-9 (**Figure 2C**). Unleashing T cell-activity by immune checkpoint blockade (ICB) therapy has been a real breakthrough in the treatment of human cancers. ICB is able to determine partial or even complete regression of primary solid tumors and metastatic lesions, for example in melanoma and lung cancer patients (22, 23). Unfortunately, only a small fraction of ICB-treated patients responds to therapy, with some variability in the percentage of responsive patients across different tumor types. Reinvigoration of tumor-antigen specific T cells and high tumor-derived neo-antigen burden have been associated to clinical response (24). The effectiveness of ICB, even though only in some of the patients, supports the concept that functional exhaustion of T cells is not an irreversible state, and/or that resetting of anti-tumor immunity can result in an effective T cell response even at advanced cancer stages. Nevertheless, a better knowledge is required to understand why the majority of patients do not respond, or experience tumor progression after an initial partial response. It is also important to understand why a few of the non-responder patients develop the so called hyper-progressive disease (HPD), which is characterized by accelerated tumor growth associated with drastic worsening of clinical conditions (25).

Anti-PD-1 and anti-PD-L1 monoclonal antibodies (mAbs) are among the most effective ICB, currently used in several tumor types (**Table 1**). PD-1 is a lymphocyte inhibitory receptor expressed by antigen-activated T cells, that binds to PD-L1 and PD-L2 expressed on the surface of other cells. PD-L1 and PDL-2 have different expression patterns; PD-L1 is expressed by several cell types, including Antigen Presenting Cells (APCs) such as macrophages and DCs, MDSCs, and tumor cells (32), whereas PD-L2 is mostly expressed by APCs and lymphocytes (33). PD-1-mediated feedback loop contributes to maintain tissue homeostasis and prevent cell damage, especially in conditions of chronic immune stimulation, e.g. chronic infections and autoimmune diseases (32). Nevertheless, PD-1 induced T-cell inhibition could be detrimental in case of anti-tumor T cell response. One mechanism of action of anti-PD-1/PD-L1 mAbs in anti-cancer treatment is to block the molecular interaction between the corresponding receptor-ligand pair, thus relieving PD-1^+^ T cells from PD-L1-mediated inhibition exerted by PD-L1^+^ tumor cells and/or myeloid cells in the tumor infiltrate (34). Additional mechanisms have been described, unraveling the complexity of the biological response to anti-PD-1/PD-L1 mAb. It has been proposed that cancer patient treatment with the PD-L1 mAb Avelumab might result in elimination of PD-L1^+^ cells by Antibody Dependent Cellular Cytotoxicity (ADCC), as suggested by *in vitro* data (30, 31); in contrast, the PD-L1 mAbs Atezolizumab and Durvalumab have an engineered Fc region to reduce ADCC (28, 29). Notably, ADCC has not been reported for the PD-1 mAbs Pembrolizumab and Nivolumab (26, 27) (**Table 1**). A detailed overview of anti-PD-1/PD-L1 mAb effects on immune response goes beyond the scope of this paper; the reader is referred to excellent recent articles on this topic (35–37).

**Table 1:** Anti-PD-1/PD-L1 monoclonal antibodies (mAbs) in anti-cancer immunotherapy.

Drug name	Target	lsotype	Degreeof humanization	Time to market	Antibody-dependent cellullar cytotoxicity (ADCC) *in vitro* activity	Cancer types
Pembrolizumab	PD-1	lgG4, κ	Fully humanized	2014	NO (26)	Non small cell lung cancer (NSCLC), melanoma, renal cell carcinoma (RCC), urothelial carcinoma, Merkel cell carcinoma, hepatocellular carcinoma, colorectal cancer (CRC), cervical cancer
Nivolumab	PD-1	lgG4, κ	Fully human	2014	NO (27)	NSCLC, small cell lung cancer (SCLC), melanoma, RCC, urothelial carcinoma, CRC, Merkel cell carcinoma, hepatocellular carcinoma
Atezolizumab	PD-L1	lgG1, κ	Humanized	2016	NO (28)	Urothelial carcinoma, SCLC, NSCLC, triple-negative breast cancer
Durvalumab	PD-L1	lgG1, κ	Human	2017	NO (29)	NSCLC, SCLC, urothelial
Avelumab	PD-L1	lgG1, λ	Human	2017	YES (30, 31)	Urothelial carcinoma, Merkel call carcinoma

The table summarizes the main features of the most common anti-PD-1/PD-L1 mAbs currently employed in anti-cancer immunotherapy.

In this article we will provide a T memory-centric vision of anti-tumor immunity. We will start with a brief outline of tumor-immune system interaction, with a focus on Tumor Infiltrating Lymphocytes (TILs). We will then give an overview of the distinct roles played by TRM and re-circulating memory T cells in antitumor immune response, and discuss emerging evidence on the effects of anti-PD-1/PD-L1 on the two types of T cells. We will propose that a better knowledge of the interplay between TRM and re-circulating memory T cells in anti-tumor immunity will provide an insightful framework to better understand the mechanisms underlying anti-PD-1/PD-L1 immunotherapy, offering new perspectives on how to improve it.

## Tumor Infiltrating Lymphocytes (TIL)

Tumor Infiltrating Lymphocytes (TILs) are a heterogeneous mixture containing tumor antigen-specific T cells, T cells of unknown specificities, Tregs, etc. (38). TILs comprise both TRM, and T cells belonging to the recirculating pool. Despite some pre-existing evidence of long-term retention of memory T cells in peripheral extra-lymphoid sites (39, 40), it was only about a decade ago that TRM were recognized as a clearly defined T cell memory subset, characterized by its own functional and molecular signature (41). Thus, in many studies TILs were examined without a separate analysis of the TRM component in them. We will briefly summarize here some of these TIL studies that lack TRM analysis, and then focus on TRM and memory recirculating T cells in the next paragraphs.

TILs have been an extraordinary tool to gain knowledge on T cell response against tumors, clone tumor antigens, and develop anti-tumor immunotherapies (42). One of the classical approaches in immunotherapy has been to re-invigorate TILs *in vitro* by treatment with IL-2, and then infuse them back into the patient (43). It was later recognized that IL-2 expanded mostly NK cells, which mediated tumor cytotoxicity upon infusion (44). Despite some success, this treatment had severe side effects, and was replaced in subsequent years by more effective tumor-tailored adoptive T cell therapies, often based on tumor-specific T cells isolated from TILs (45–47).

TILs contain cytotoxic CD8 T cells and helper CD4 T cells, as well as Tregs. When a tumor reaches a clinically evident stage, TILs have predominantly a terminally differentiated phenotype, but they somehow failed to clear the tumor. Indeed, they are inhibited by a variety of immunosuppressive mechanisms in the TME, including inhibition by high levels of TGF-β and/or other cytokines, negative regulation by innate cells such as Tumor Associated Macrophages (TAM) and MDSCs, metabolic competition with tumor cells (48), and an imbalance among T cell subsets, with a Treg dominance (49). It has been proposed that TIL exhausted phenotype is under the control of the transcription factor TOX (50, 51), nevertheless TOX has been also implicated in terminal differentiation of effector T cells, thus questioning its exhaustion-specific expression (52, 53). It should be noted that “exhaustion” is a comprehensive term which includes different T cell-phenotypes described in diverse experimental models, as mentioned above (21). A likely scenario is that a set of transcription factors (e.g. Eomes, T-bet, TCF-1, TOX, etc.) jointly regulates effective and dysfunctional T cell differentiation, as suggested by experimental findings in mouse models (54–56). Advanced technologies, including multidimensional flow cytometry, TCR sequencing and single cell -omics, have greatly contributed to our growing understanding of TIL heterogeneity (57–59). Patient-derived organoids from tumor biopsies are a new highly promising tool to investigate TILs embedded in their original TME, and gain information on the functional and/or exhausted profile of TIL subsets (60).

Anti-PD-1/PD-L1 treatment is aimed at unleashing T cell response against the tumor, thus it is expected that in responder patients TILs change following treatment, for example their number might increase due to recruitment of T cells from circulation and/or local proliferation. In fact, TIL quantification and characterization are considered among the “dynamic” biomarkers of response to ICB, which can be examined in tumor biopsies (49). Experimental studies performed with single cell approaches have been recently used to track changes occurring in TILs in response to ICB (61). One of these studies has questioned that all TILs are exhausted in advanced tumors, and has proposed instead that even without treatment a few are functional (e.g. those expressing TCF-1), and they are simply expanded by ICB (62), in agreement with the proposed role of TCF-1 in mouse models of chronic infections (63). Once again, reaching a consensus on the definition of exhaustion might help to solve some current discrepancies (21).

Changes in the Treg fraction of TILs have been associated with clinical response to ICB. About 10% of gastric cancer patients treated with anti-PD-1 experience HPD. In these patients, Tregs with effector phenotype (FoxP3^high^CD45RA^—^ CD4 T cells) expressing the cell cycle marker Ki-67 increased among TILs after treatment, whereas in non-HPD patients these cells diminished, suggesting that Treg expansion in TME supports increased local immunosuppression and disease worsening (64).

## Tissue-Resident Memory T Cells (TRM)

TRM characterization is essential for a full evaluation of T cell response in TME. This subset of non-migratory memory T cells was identified about 10 years ago, in the course of seminal studies on peripheral immune defense against viral infections, which focused on CD8 TRM (5, 65). It was shown that CD8 T cells recruited to the skin upon Herpes Virus infection generated a population of skin-resident memory CD8 T cells that did not go back to circulation and controlled viral growth locally (65). CD4 TRM have been subsequently identified, and are currently under intense investigation in diverse settings (66). TRM can be found in many epithelial barriers (i.e. skin, lung, gastroenteric and reproductive tracts) and also in internal organs (e.g. kidney, brain) to ensure long-term immunity against infections, tumors and other types of tissue damage (5, 67–69). TRM have a remarkable capacity for exerting protective functions, e.g. cytokine production, etc. (5).

A distinct set of surface molecules is typically expressed by TRM, including the adhesion molecule CD103 (the αE integrin subunit) and the activation marker CD69 (70), nevertheless there are also CD103^—^ TRM (5, 6). Notably, TRM differ from other T-memory subsets, such as TCM or TEM, in terms of transcription profile, metabolism, kinetics of response, and migration capability into extracellular matrix (71, 72). The signals required for priming and differentiation of TRM have been only partially disclosed. For example, it is unclear whether αE/β7 integrin interaction with E-cadherin, which is highly expressed in epithelial tissues, provides survival and/or other signals to CD103^+^ TRM, and what are the differences between CD103^+^ and CD103^—^ TRM (5). Moreover, it has been shown that DCs expressing DNGR-1 (the C-type lectin receptor for F-actin) are required for optimal priming of TRM but not of recirculating T cells in a mouse model of viral infection (73). The degree of plasticity of antigen-experienced effector and memory T cell subsets to generate TRM remains to be determined (74). It should be noted that perturbation of normal T cell traffic is required to definitely identify TRM, that by definition are non-recirculating cells; this question is normally addressed in mouse models.

Recent evidence indicated that human CD8^+^ TILs from epithelial cancers contain TRM-like cells (i.e. cells expressing TRM markers) and that their abundance is associated with strong anti-tumor activity (75). For example, Guo and colleagues performed single-cell sequencing analysis of TILs within Non-Small-Cell-Lung Cancer (NSCLC) specimens, and identified several intratumoral CD4 and CD8 T cell clusters, including TRM-like cells expressing high levels of mRNA coding for CD69, for the chemokine receptor CXCR6, and for the integrins CD49a (ITGA1 gene), and CD103 (ITGAE gene) (76). Studies in lung cancer showed that patients with greater intratumoral density of TRM-like cells had a better prognosis (77). Similarly, high intratumoral frequency of CD103^+^ CD39^+^ CD8^+^ T cells was associated with better overall survival in patients with head-and-neck squamous cell carcinoma, another type of epithelial cancer (78). Recent findings in mouse models suggest that TILs with CD69^+^ CD103^+^ TRM-like phenotype are found also in non-epithelial cancers, such as rhabdomyosarcoma (79).

Since TRM may express different inhibitory receptors, this subset represents a potential target for ICB (80, 81). A preferential expression of PD-1 and TIM-3 by intratumoral TRM-like cells has been observed in lung, cervical, ovarian, endometrial cancer and melanoma, both in mice and humans (82). Remarkable changes in TRM-like cells have been documented in patients responding to ICB (83–85). In one of these studies, a positive response of NSCLC patients to anti-PD-1/PD-L1 therapy was correlated to an increased intratumoral density of CD8^+^ CD103^+^ TILs, which displayed typical transcriptomic and phenotypic profiles of TRM (83). Similarly, in anti-PD-1-treated lung cancer patients, it was observed that CD8^+^ CD103^+^ TILs accumulated in patients with better progression-free survival; these TILs were enriched with TRM-like cells having a unique Tc1/Tc17 effector signature, further emphasizing the distinguished differentiation program of TRM and their critical role in response to ICB (84). TCR sequencing studies in melanoma showed that TRM-like clones were diverse in different metastatic lesions from the same patient, with implications for heterogeneity of ICB-induced unleashing of anti-tumoral activity at each site (80)

## Recirculating Memory T Cells

Recirculating memory T cells are found in the lymph and in the peripheral blood, and migrate in and out of lymph nodes, spleen, BM, and extra-lymphoid tissues, thus patrolling the whole body to provide systemic protection. Upon tumor antigen-recognition, recirculating memory T cells can develop highly efficient secondary responses, resulting in tumor cell killing, cytokine release, etc. In a study on mouse melanoma, it has been shown that CD8 mAb treatment inducing 82%–99% reduction of circulating CD8 T cells resulted in rapid metastasis outgrowth in visceral organs, suggesting that CD8 T cells were cytostatic and kept in check disseminated dormant tumor cells in this model (86). Conversely, there are some rare cases of T cells favoring the metastatic process. For example, T cell pro-osteoclastogenic activity can favor bone erosion and remodeling, supporting breast cancer cell metastatization to the bones (87). Recirculating T cells migrating to tissues distant from primary tumor are likely to be involved in this case, nevertheless TRM contribution was not investigated and cannot be excluded.

Peripheral blood samples from cancer patients have been extensively screened for the presence of tumor antigen-specific T cells, that have been identified and characterized in a number of patients (88–90). In some cancer patients at advanced stages, TCR repertoire skewing and impairment of peripheral blood T cell function have been observed (91). For example, in breast, lung and cervical cancers a decreased TCR diversity correlated with reduced capacity of IFN-γ and IL-2 production by peripheral CD4 and CD8 T cells (92, 93). Furthermore, T cell signaling defects have been reported in individuals with advanced cancers (94).

Notably, tumor antigen-specific T cells recirculate in the bone marrow (BM), and it has been shown that they are enriched in this organ as compared to peripheral blood in many patients with solid tumors, for example in subjects with melanoma and pancreatic cancer (88, 89). This is perhaps not surprising, considering that the BM has a central role in long-lived memory T cell maintenance in a variety of settings (7, 95). In solid cancer patients it cannot be excluded that BM T cells are actively engaged in micrometastasis control in this organ, even in the absence of evident metastases. BM tumor-specific T cells are functional, for example they produce IFN-γ and TNF-α (89, 96), and in most cases they are not inhibited by Tregs in the organ (97, 98). Considering that the BM represents a reservoir of functional memory T cells in tumor-bearing individuals, innovative anti-tumor T cell transfer approaches exploiting the BM as a source of T cells have been proposed (99–101). A related strategy is based on the adoptive transfer of CXCR4-engineered T cells with increased homing to the BM (102).

The re-invigoration of recirculating T cells induced by ICB has been investigated in mouse models of chronic infections and tumors, focusing on exhausted T cells. In LCMV chronic infection, the prototypical model of T cell exhaustion, it has been observed that ICB-induced functional CD8 T cells derived from rare CXCR5^+^ CD8 T cell precursors in lymphoid organs that shared molecular signature with follicular helper CD4 T cells and hematopoietic stem cell progenitors (103). Studies in other mouse models implicated T cell-intrinsic CD28 expression in the proliferative CD8 T cell response to PD-1 blockade, suggesting that engagement of the CD28/B7 co-stimulatory pathways, possibly occurring in lymphoid organs, has a central role in response to treatment (104).

Remarkably, in many human studies, distinct changes of peripheral blood T cells following PD-1/PD-L1 blockade have been associated with response to treatment. For example, it has been shown that Ki-67^+^ CD8 T cells appeared in peripheral blood of lung cancer patients treated with anti-PD-1, suggesting that these cells switched from a quiescent to an activated/proliferative state and were mobilized in the circulation (104, 105). Functional memory CD4 T cells in peripheral blood at baseline and increased proportions of Ki-67^+^ CD4 T cells after anti-PD-1/PD-L1 have been associated with better responses to treatment in NSCLC patients (106). These changes in peripheral blood T cells can potentially be exploited as biomarkers of response.

The intra-tumoral recruitment of recirculating T cells upon anti-PD-1/PD-L1 treatment has been investigated in depth by a few reports. In a big transcriptomic study, in which >300 million T-cell derived mRNA transcripts were sequenced, the same expanded clonotypes of T cells were found in the tumor, in normal adjacent tissue, and in peripheral blood, thus suggesting that non-exhausted recirculating T cells from non-tumoral sites are recruited into the tumor in response to anti-PD-L1 (107). Replacement of intratumoral T cell clones with newly recruited T cells upon PD-1 blockade therapy has been shown in basal and squamous cell carcinoma (108). It is tempting to speculate that the newly recruited T cells may include memory T cells switching to an effector phenotype in the tumor bed upon local restimulation with antigen.

## The Emerging Division of Labor Between TRM and Recirculating Memory T Cells in Anti-Tumoral Immunity

That local and systemic anti-tumor immunity might be discordant has long been known. One example is the phenomenon of concomitant immunity, that was described some decades ago in transplantable tumor models, when it was shown that an individual bearing a primary growing tumor rejected a secondary syngeneic tumor at a distant site (109). Rejection was T-cell mediated and occurred only at early times after primary tumor inoculation, before the growing tumor evoked a population of suppressor T cells that inhibited anti-tumor response in the whole body (110). These old experiments were then revisited more recently, as there was a resurgent interest for Tregs (111, 112). It would be interesting to reconsider concomitant immunity in the light of the dichotomy between TRM and recirculating memory T cells. For example, one can envision that in the old experiments with transplantable tumors, the sensitizing primary tumor induced antigen-specific recirculating T cells, but was not seeded by local TRMs, resulting in incomplete anti-tumor protection. This experimental model might resemble human cancers that are resistant to T-cell infiltration.

As concerns T-cell infiltrated tumors, it is conceivable that tumor antigen-specific TRM, that permanently reside in the tumor bed and are chronically antigen-exposed, display more evident signs of exhaustion than recirculating memory T cells, which are intermittently exposed to tumor-derived antigens, e.g. in tumor-draining LNs, as suggested by studies on TRM-like cells in human urinary bladder cancer (113). Furthermore, many other factors in TME can promote T-cell exhaustion locally, including infiltrating Tregs, MDSCs, inhibitory cytokines, etc., as discussed above. In this context, migration of recirculating tumor-specific T cells to the BM might sustain their persistence and functionality, in agreement with the supporting role of the BM in long-term memory (7). From this organ, tumor-specific recirculating memory T cells can be mobilized into the blood and recruited into the tumor to exert their protective activity (7, 100).

**Figure 3:**
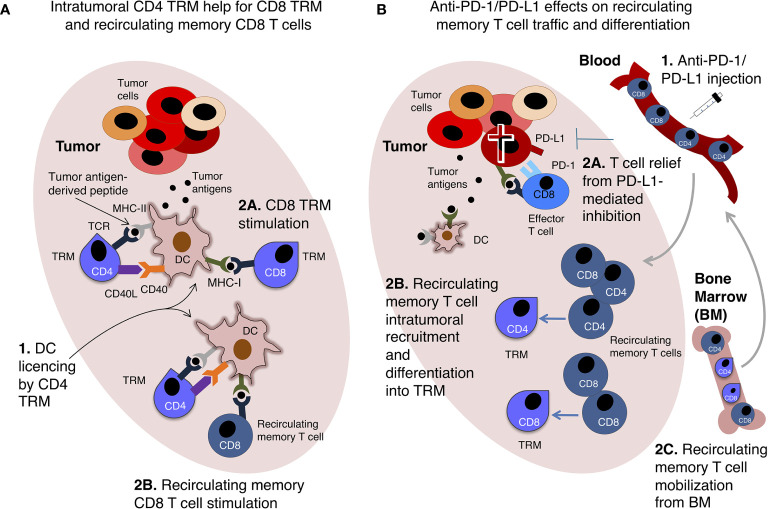
Tissue-resident memory T cells (TRM) and recirculating memory T cell collaboration in anti-tumor immunity. Two examples of possible interplay between TRM and recirculating memory T cell are shown. **(A)** Intratumoral CD4 TRM provide help to recirculating memory CD8 T cells and CD8 TRM. A hypothetical scenario of intratumoral DCs presenting tumor antigen-derived peptides to memory CD4 and CD8 T cells is shown. In this scenario, memory CD4 T cells are intratumoral TRM, and license DCs *via* CD40L-CD40 interaction (1). This enables DCs to fully stimulate either CD8 TRM (2A) or recirculating memory CD8 T cells (2B). **(B)** Recirculating memory T cells unleashed by anti-PD-1/PD-L1 therapy promote intratumoral TRM response. Upon anti-PD-1/PD-L1 intravenous injection (1), tumor-specific effector CD8 T cells expressing PD-1 are relieved from PD-L1-mediated inhibition and kill tumor cells **(2A)**. Recirculating memory CD4 and CD8 T cells migrate *via* blood into the tumor, and differentiate into TRM **(2B)**, augmenting TIL number and anti-tumoral activity. Recirculating memory CD4 and CD8 T cells are mobilized from BM into the blood, and are then recruited into the tumor, thus contributing to re-invigorate anti-tumor T cell immunity **(2C)**. These hypothetical examples echo data obtained in mouse models of immune response against viruses **(A)** (116) and transplanted tumors **(B)** (117).

Conversely, TRM may have an advantage over recirculating memory T cells because of their interaction with distinct types of intratumoral DCs. Indeed, it has been shown that CXCR6^+^ T cell contact with CCR7^+^ DC expressing the CXCR6 ligand CXCL16 and transpresenting IL-15 supported T cell survival in intratumoral perivascular niches (114). In this context, CXCR6 up-regulation could represent a transitory rescue signal for terminally differentiated or exhausted T cells (114). Furthermore, considering that most secondary CD8 T cell responses rely on CD4 T cell help (115), which is mediated by the antigen-presenting DC (1–4), it is conceivable to envision that CD4 TRM can license intratumoral DCs for productive restimulation of TRM and/or recirculating memory CD8 T cells infiltrating the tumor (**Figure 3A**). This possibility is consistent with recent findings showing that CD4 TRM provide help for memory CD8 T cells in antiviral immune responses in the lungs (116).

## TRM and Recirculating Memory T Cells in PD-1/PD-L1 Blockade and Combination Therapies

Beyond the well-established specialization of TRM and recirculating memory T cells in providing local and systemic protection, respectively, an insightful mouse study proposed that PD-1/PD-L1 blockade can strengthen their interplay. Indeed, anti-PD-1 treatment promoted intratumoral infiltration of intravenously transferred tumor antigen-specific TCM, without increasing the numbers or frequency of these cells in tumor-draining LNs (117). In this study, adoptively transferred tumor antigen-specific TCM showed potential to give rise to TRM-like cells upon tumor inoculation (117). These findings point to the developmental plasticity of memory T cells, a topic deserving further investigation for a better understanding of T cell response to ICB. Furthermore, building on these results (117), it can be envisioned that recirculating memory T cells recruited into the tumor after anti-PD-1/PD-L1 are enriched in recently mobilized BM T cells (**Figure 3B**).

Since PD-1/PD-L1 blockade is a systemic treatment, it can have a broad effect on T cell responses, beyond those on tumor-specific T cells. Peripheral blood T cells are probably more informative than TRM about the potential re-invigoration of T cells specific for non-tumoral antigens, and/or cross-reactive T cells, occurring in cancer patients after ICB (118). Early identification of this phenomenon might be important to reduce the potential risks of immune-related Adverse Events (irAEs) due to activation of auto-reactive T cells (49, 119). Conversely, cross-reactivity of T cell clones might be exploited against tumor cells. For example, it has been shown that some memory T cells in peripheral blood specific for melanoma antigens were able to recognize also antigens of common pathogens such as Herpes Simplex Virus-1, and *Mycoplasma penetrans* (120, 121).

Ideally, anti-PD-1/PD-L1 should reset both local and systemic T cell reactions against tumor cells, resulting in effective tumor elimination, and long-term prevention of recurrence and/or metastases. Unfortunately, only some patients benefit of this therapy. To explain the mechanisms underlying anti PD-1/PD-L1 resistance, it has been proposed that tumors are “cold” in non-responder patients, with reduced T cell infiltration, lack of tumor antigens, defect in antigen presentation, or presence of mechanisms blocking T cell migration into the tumor site (122). Of note, intratumoral flu vaccination is able to transform immunologically “cold” tumors into “hot” tumors, and in combination with ICB is highly effective against mouse melanoma (123). It is also possible that anti-cancer T cell response is either quantitatively or qualitatively inadequate to effectively eliminate the tumor, in at least some of the patients who do not respond to anti-PD-1/PD-L1. Thus, these patients would better benefit of anti-cancer vaccination, and/or other strategies aimed at boosting proinflammatory (Th1) CD4 T cells and cytotoxic CD8 T cells (34). To increase the proportion of responder patients, combinations of anti-PD-1/PD-L1 and conventional chemotherapy have been tried. A significant improvement of therapeutic response has been reported in advanced human NSCLC and renal cell carcinoma treated with anti-PD-1/PD-L1 mAbs combined with standard chemotherapics (124–126). Chemotherapics may trigger immunogenic tumor cell death, resulting in stronger antigen presentation and co-stimulation, tumor-specific T-cell activation/traffic and tumor cell destruction (127, 128). A comprehensive discussion of the many combination ICB therapies, already in use or at different stages of development, and of their proposed underlying mechanisms, goes beyond the scope of this review. We would only propose here that combining ICB with drugs strengthening a productive collaboration between TRM and non-migratory memory T cells might open new avenues for cancer immunotherapy.

## Concluding Remarks

The concerted anti-tumoral action of TRM and recirculating memory T cells may be required for efficient and durable protection, and for response to anti-PD-1/PD-L1(**Box 1**). However, the role of T cell migration and residency in anti-tumor response has not been fully investigated and many questions remain still open in the field (**Box 2**). Multi-organ analysis could provide critical information on the contribution of the two types of T cells to either protective or pro-tumorigenic mechanisms in tumor-bearing hosts (129). We would like to stress that, among other mechanisms, failure of T cell response in the majority of anti-PD-1/PD-L1-treated cancer patients might derive from insufficient re-invigoration of either TRM or recirculating memory T cells, and from a non-productive interplay between the two types of T cells (**Figure 3**). It is tempting to suggest that revising the “Cancer immunoediting” concept to take into consideration the migratory behavior of anti-tumoral T cells might contribute to achieve a more comprehensive view of anti-PD-1/PD-L1 mechanism of action, and consequently to improve anti-cancer therapy, for example by combining ICB with drugs able to modulate T-cell homing pathways.

Box 1 **Key Points.**

• It is still poorly understood how local and systemic T-cell immunity collaborate in anti-tumor response, before and after the administration of anti-PD-1/PD-L1   mAbs.
• Tumor-infiltrating T cells (TILs) comprise both tissue-resident memory T cells (TRM), which are non-migratory T cells that permanently reside in the tumor, and   recirculating memory T cells, that can be recruited into the tumor.
• TRM are constantly exposed to local signals, mostly immunosuppressive, in the Tumor Microenvironment (TME).
• Recirculating memory T cells can be recruited to the tumor site after anti-PD-1/PD-L1 mAbs treatment.
• Migration of recirculating tumor-specific T cells to the bone marrow (BM) might support their persistence and functionality; from this organ they can be mobilized   into the blood and recruited into the tumor.
• Multi-organ analysis could be highly informative about the contribution of different T cell subsets to either protective or pro-tumorigenic mechanisms.
• We propose to revise the “Cancer immunoediting” concept to take into consideration the migratory behavior of anti-tumoral T cells, to achieve a comprehensive   view of TRM and recirculating memory T cell response to solid cancers before and after anti-PD-1/PD-L1 mAb treatment.

Box 2 **Open Questions in the Field.**

• Which is the role of tissue-resident memory T cells (TRM) and recirculating memory T cells in anti-tumor response before and after anti-PD-1/PD-L1 therapy?
• Are recirculating memory T cells exposed to less immunosuppressive environments compared to intratumoral TRM (e.g. in the bone marrow)?
• Can TRM and recirculating memory T cells influence the immune cell composition of the tumor infiltrate?
• Which is the role of environmental factors, infectious agents or microbiota in anticancer TRM response before and after anti-PD-1/PD-L1 therapy?
• Which is the role of environmental factors, infectious agents or microbiota in anticancer recirculating memory T cell response before and after anti-PD-1/PD-L1  therapy?

## Author Contributions

SG wrote the initial draft of the manuscript. AN and FA edited the manuscript. FD revised the final version of the manuscript. All authors contributed to the article and approved the submitted version.

## Funding

Grant sponsor: Italian Minister of Research and University (MIUR), grant number: 2017K55HLC.

## Conflict of Interest

The authors declare that the research was conducted in the absence of any commercial or financial relationships that could be construed as a potential conflict of interest.

## Publisher’s Note

All claims expressed in this article are solely those of the authors and do not necessarily represent those of their affiliated organizations, or those of the publisher, the editors and the reviewers. Any product that may be evaluated in this article, or claim that may be made by its manufacturer, is not guaranteed or endorsed by the publisher.
